# Streamlined spectrofluorimetric assay for Pramipexole using acid red 87 quenching strategy for tablet and uniformity evaluation

**DOI:** 10.1038/s41598-025-24218-x

**Published:** 2025-11-07

**Authors:** Ahmed A. Abu-hassan

**Affiliations:** Department of Pharmaceutical Analytical Chemistry, Faculty of Pharmacy, Assiut Branch, Al-Azhar University, Assiut, 71524 Egypt

**Keywords:** Acid red 87, Spectrofluorimetric, Ion-pair, Tablets, Assay, Chemistry, Analytical chemistry

## Abstract

Pramipexole (PPX) is prescribed for alleviating parkinson’s disease symptoms like tremors and mobility issues, as well as addressing the discomfort in the legs and sudden leg movement impulses associated with restless legs syndrome. This study presents a practical, direct, non-extractive, and cost-effective analytical strategy for the quantitative determination of PPX. The method leverages fluorimetric detection, specifically monitoring the quenching effect on the emission intensity of Acid red 87, a fluorone dye. Complexation between PPX and Acid Red 87 occurs rapidly within a single reaction vessel under acidic conditions. A linear relationship was established between the observed decrease in Acid red 87 fluorescence intensity and PPX concentration, enabling the construction of a calibration curve spanning 50 to 1400 ng/mL. Critical variables influencing the PPX-dye complex system were systematically investigated and optimized. Comprehensive validation studies, conducted in accordance with International Council for Harmonisation (ICH) guidelines, confirmed the method’s reliability. The validated approach was subsequently applied successfully to assay PPX in pharmaceutical tablets and to evaluate tablet content uniformity.

## Introduction

 Pramipexole ((6*S*)−6-*N*-propyl-4,5,6,7-tetrahydro-1,3-benzothiazole-2,6-diamine M.wt = 211.33 g·mol^−1^) is a medication utilized in managing Parkinson’s Disease manifestations. As a non-ergot dopamine agonist, it proves effective in addressing a range of Parkinson’s symptoms like tremors, rigidity, and bradykinesia. With both dopaminergic and neuroprotective properties, it emerges as a hopeful option for bipolar depression treatment^1^. PPX acts as a presynaptic agonist, displaying an 8-fold higher affinity for D3 receptors compared to D1, D2, and D4 receptors. The D3 receptor, which is prominently present in the mesolimbic system, is associated with the anhedonic and motor aspects of depression^2^. The first negative effects of pramipexole resemble those seen with other dopamine agonists and levodopa, including nausea, vomiting, and orthostatic hypotension. Peripheral oedema, sleeplessness, and hyperalgesia are prevalent as well^3^.

The literature review on PPX indicates that its determination has been achieved through various methodologies, including spectrophotometry^4–14^, spectrofluorimetry^14,15^, chemiluminescence^16^, TLC-densitometry^17^, HPLC^10,18–25^, LC-MS^26–30^, ultrafast liquid chromatography^31^, electrochemical techniques; voltammetry^32–37^, and capillary electrophoresis^38^.

The majority of existing methods rely on chromatography, necessitating costly equipment, significant amounts of hazardous and ultrapure solvents, and complex and time-intensive sample preparations. Moreover, many of these methods utilize expensive mass spectrometry detectors. Spectrophotometric approaches often suffer from subdued sensitivity, occasionally requiring harmful reagents or solvents. Conversely, spectrofluorometry offers a straightforward and sensitive means of quantifying various pharmaceutical compounds accurately and precisely using user-friendly equipment. However, only two spectrofluorometric techniques have been documented for determining PPX, albeit involving a lengthy process using NBD-Cl derivatization at high temperatures. The other reported approach used the expensive reagent fluorescamine.

Xanthene dyes and related compounds are garnering heightened scientific interest owing to their substantial utility in analytical chemistry, pronounced binding affinity for biomolecules, and high fluorescence quantum yields. Acid Red 87, a halogenated fluorone derivative, exemplifies this class and finds application as a laser dye, food colorant, and agent in medical diagnostics. Its established capacity for preferential protein binding, coupled with inherent photoluminescence, further validates its role as a sensitive reagent for protein quantification within biological matrices^39^. Consequently, Acid Red 87 is recognized as a promising fluorogenic probe for bioanalysis. This utility extends to the quantitative determination of several pharmaceuticals, including asenapine^40^, atomoxetine^41^, eletriptan^42^, dothiepin^43^, and mebeverine^44^. The present work demonstrates the effective application of Acid Red 87 as a fluorogenic probe for the practical quantification of PPX. Rigorous validation according to International Council for Harmonisation (ICH) guidelines confirmed the method’s robustness. The validated procedure was successfully implemented for assaying PPX in pharmaceutical tablets and assessing content uniformity. An additional significant advantage is the method’s environmental compatibility; utilizing distilled water as the solvent medium aligns with the core principles of green chemistry.

## Experimental

### Apparatus

The test samples were analyzed for fluorescence using an FS-2 Spectrofluorimeter (Scinco Co., Seoul, Korea), within a 1-cm quartz cell. Illumination was sourced from a 150 W xenon arc lamp. The photomultiplier tube (PMT) integration time was established at 50 ms, and all scans were executed at a rate of 578 nm per minute. Data acquisition was achieved via software FluoroMaster Plus v2.1 (Scinco Co.). Additionally, a pH meter (Adwa Instruments, Romania) was employed during the experimental procedures. Equipment such as the SC-101TH bath sonicator (Sonics & Materials Inc., USA) was also used.

### Chemicals and materials

All of the compounds that were utilized in the research adhered to the necessary analytical grade and quality standards. The original pramipexole sample was obtained from Eva Pharma in Egypt, without any further processing. Ramixole tablets containing 1.0 mg of PPX per tablet (Batch No: 6223002450633) were purchased from a community pharmacy in Tanta. To prepare a 0.03 w/v Acid red 87 solution, 30 mg of the compound from Sigma in Missouri, USA, was initially dissolved in a small quantity of water. The resulting solution was then diluted in a 100 mL volumetric bottle and adjusted to mark with distilled water. El-Naser Chem. Co. in Egypt provided the acetonitrile, dioxane, acetone, dimethylformamide, ethyl, isopropyl, methyl alcohols, and sodium hydroxide used in the experiments.

### Stocks solutions

#### Standard PPX solution preparation

A primary stock solution of PPX (100 µg/mL) was prepared by quantitatively transferring 10 mg of PPX into a 100 mL volumetric flask. The compound was dissolved in a minimal volume of distilled water before diluting to the final volume with the same aqueous medium. Working standard solutions at various concentrations were subsequently prepared by appropriate volumetric dilution of the stock solution using distilled water. All solutions were stored at 4 °C to ensure chemical stability until analysis^45^.

#### Teorell-Stenhagen buffer system

To achieve precise pH control across the operational range of 2.0 to 12.0, a Teorell-Stenhagen buffer system was employed. This system utilized two master solutions. The first contained a mixture of phosphoric acid, citric acid, and sodium hydroxide. The second master solution consisted of 0.1 M hydrochloric acid. The desired operational pH was attained by accurately mixing specific proportions of these two master solutions.

### Execution of analytical procedures

Calibration standards encompassing the PPX concentration range of 50 to 1400 ng mL⁻¹ were prepared by transferring precisely measured 1.0 mL aliquots of the respective standard solutions into individual 10 mL volumetric flasks. To each flask, 1.2 mL of Acid red 87 reagent solution and 1.3 mL of Teorell-Stenhagen buffer (pH 3.8) were added. The mixture was gently swirled to ensure homogeneity and allowed to react for 5 min. Subsequently, the solution was diluted to the 10 mL mark with distilled water. A reagent blank solution was prepared identically, but omitting the PPX solution. The quenching effect of PPX on the inherent fluorescence intensity of Acid red 87 was then quantified fluorometrically. Measurements were performed at an emission wavelength of 546.8 nm following excitation at 302.8 nm.

### Tablets analysis

For the assay of pharmaceutical formulations (Ramixole 1 mg tablets), a representative number of tablets was accurately weighed and collectively triturated to a homogeneous fine powder. A portion of this powder, equivalent to 10 mg of PPX, was quantitatively transferred into a 100 mL volumetric flask. Aqueous extraction of the PPX active pharmaceutical ingredient (API) was performed using distilled water as the solvent. The resulting solution was filtered, typically through a cellulose membrane filter (e.g., 0.45 μm), to remove insoluble excipients before analysis. The filtrate was then appropriately diluted, and the PPX concentration was determined using the validated fluorometric method. The assay results were calculated based on the established calibration regression equation. Content uniformity assessment for Ramixole 1 mg tablets was conducted according to pharmacopeial standards by performing individual tablet assays on ten separate tablets, following the identical preparation and analytical procedure for each unit^46–48^.

### Result and discussion

Xanthene dyes and their structural derivatives continue to attract significant research interest within analytical chemistry, primarily due to their intrinsic capacity to interact with biomolecules and their consequent analytical utility^49,50^. Acid Red 87 exemplifies this class, serving as a valuable fluorone-based chemical probe where halogen atoms substitute the core structure. Its application in detecting basic pharmaceuticals hinges on a characteristic reaction: interaction with amino group-containing drugs generates a novel chromogenic species^40^. This binding event often manifests through detectable changes in optical properties, including enhanced Rayleigh scattering signals^51^ or, more commonly, a measurable quenching of the dye’s characteristic fluorescence emission^42^. The versatility and robust analytical performance demonstrated by Acid Red 87 underscore the broader value of xanthene dyes in drug analysis and detection methodologies.

Specifically, the quantifiable fluorescence quenching response observed upon tetrabromofluorescein’s interaction with protonatable basic drugs provides a sensitive foundation for determining their concentration. This principle has been successfully applied to quantify therapeutic agents such as fluvoxamine^41^, eletriptan^42^, and certain β-blockers^52^. The analytical scope of this fluorescence quenching technique extends considerably beyond these documented examples, suggesting potential applicability to a wider array of basic pharmaceuticals and related compounds capable of analogous interactions with Acid Red 87 or similar xanthene derivatives.

**Fig. 1:**
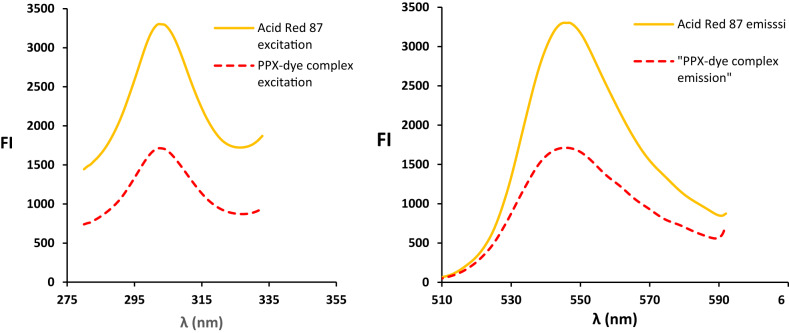
Excitation and emission spectra of the Acid red 87 dye and its reaction product with PPX (800 ng/mL).

Spectroscopically, Acid Red 87 exhibits a distinct natural fluorescence, with an emission maximum at 546.8 nm following excitation at 302.8 nm. However, this characteristic emission is markedly diminished upon introduction of a basic compound like PPX, as depicted in Fig. 1. Crucially, the extent of this quenching effect correlates quantitatively with the PPX concentration, forming the basis for analytical determination.

**Fig. 2:**
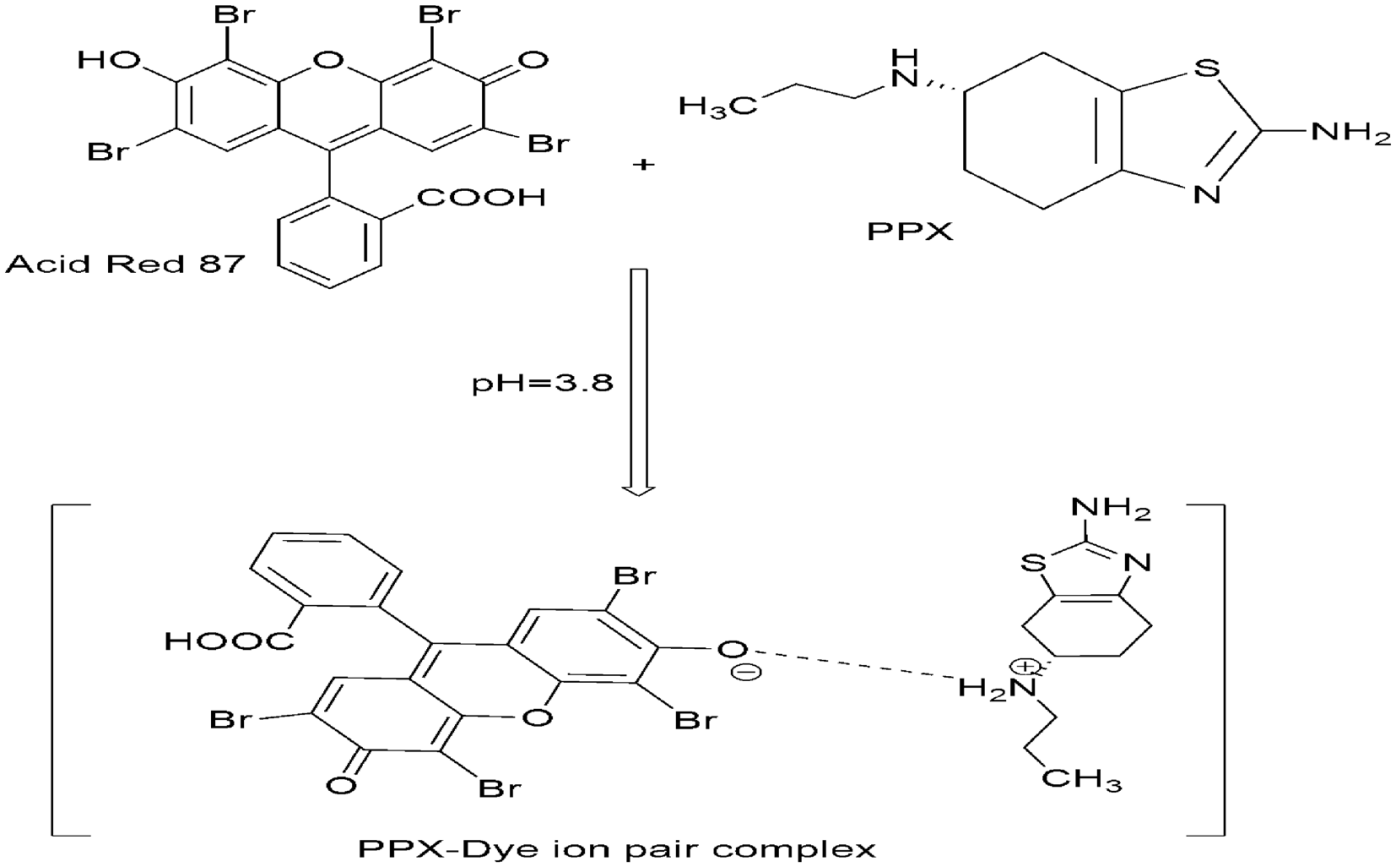
the pathway for ion-pair formation between PPX and Acid Red 87.

The underlying mechanism involves the formation of a 1:1 ion-associate complex **(**Fig. 2**).** This complexation is initiated when the drug molecule, protonated at its secondary amino group under mildly acidic conditions to form a cation (PPX⁺), encounters the anionic form of the dye. Acid Red 87 undergoes dissociation in this environment, predominantly yielding a monovalent anion. Although possessing both carboxylic acid and phenolic hydroxyl groups, ionization occurs preferentially at the hydroxyl group situated on the xanthene nucleus. This preference arises from the electron-withdrawing influence exerted by the adjacent bromine atoms, which enhances the acidity of the hydroxyl proton relative to the carboxylic acid group. Consequently, the resulting Acid Red 87 anion (predominantly from -OH ionization) readily associates with the protonated drug cation via electrostatic attraction. The stability of the resulting complex is further reinforced by hydrophobic interactions between nonpolar regions of the two molecules.

A notable advantage of this analytical approach is the rapid kinetics of complex formation between PPX and the dye, leading to shorter assay durations and enhanced procedural simplicity. Furthermore, Acid Red 87 offers a favorable safety profile compared to many alternative fluorogenic reagents and complexing agents. This characteristic aligns well with the growing emphasis on green chemistry principles within modern analytical practice. Importantly, the methodology avoids labor-intensive and environmentally concerning steps, such as hazardous solvent extraction or thermal treatment of samples.

### Conditions study and adjustment

Numerous tests were undertaken to improve the spectroscopic methodology suggested in this research. The experiments aimed to evaluate and optimize several reaction variables that can influence the outcomes.

### pH and buffer volume

**Fig. 3:**
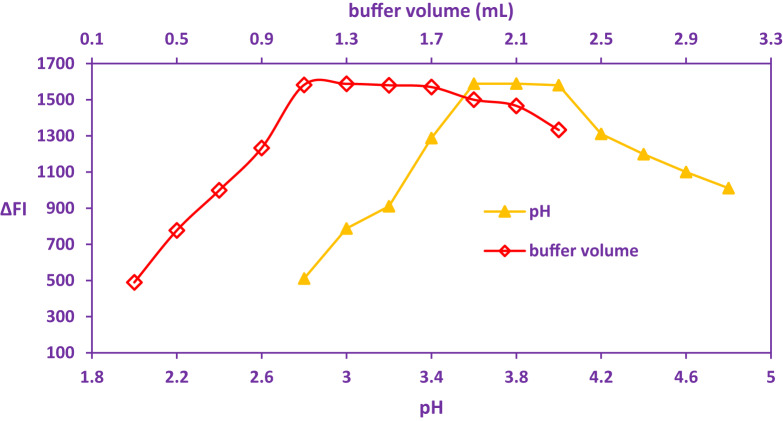
Impact of various pH and buffer volumes in PPX-Acid red 87 Dye complex formation using PPX (800 ng/mL).

Spectroscopic evaluation of the ion-pair complexation process between PPX and Acid Red 87 revealed a significant dependence on medium acidity. The formation and stability of the PPX- Acid Red 8 ion-associate complex were demonstrably influenced by pH, with maximal complex formation observed within the acidic range of pH 3.6 to 4.0, as evidenced by the data presented in Fig. 3. This pronounced pH sensitivity underscores the critical role of precise pH adjustment in optimizing the analytical signal. Based on the experimental outcomes, an optimal pH of 3.8 was selected for subsequent investigations to ensure maximal analytical response.

Furthermore, the influence of TS buffer volume on complex development was systematically investigated using the proposed spectral method, testing volumes from 0.3 to 2.3 mL. The results indicated that the volume of buffer employed exerted a significant impact on the efficiency of complex formation. The most favorable analytical responses for the methodology were consistently achieved within a buffer volume range of 1.1 to 1.7 mL. Deviations from this optimal range resulted in a measurable attenuation of the response signal. Higher buffer volumes elevated ionic strength due to increased salt concentration, which impeded the electrostatic interaction between the cationic PPX species and the anionic Acid Red 87 ligand, thereby hindering complex generation. Conversely, adequate buffer volume proved sufficient for achieving and maintaining the requisite pH environment. Consequently, a TS buffer volume of 1.3 mL, representing the midpoint of the optimal range, was adopted for this study, as illustrated in Fig. 3.

### Acid red 87 volume

**Fig. 4:**
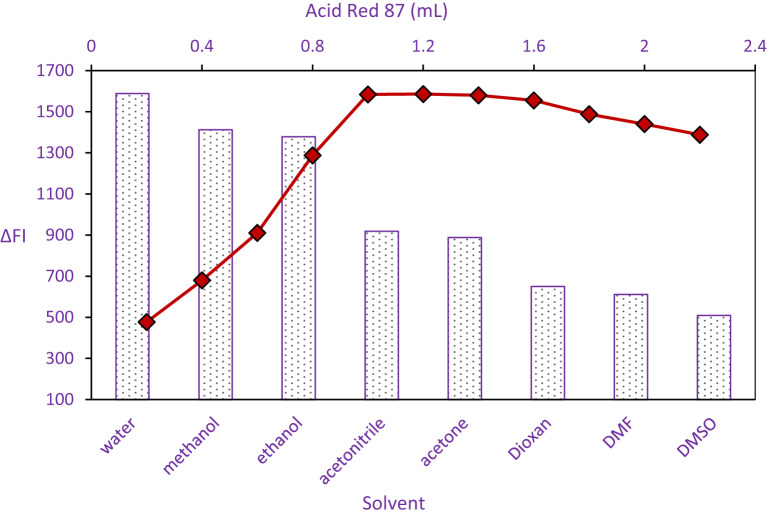
impact of Acid Red 87 dye volume and various dilution solvents on PPX-Dye formation using PPX (800 ng/mL)..

To optimize the complexation reaction, the study systematically evaluated varying concentrations of the Acid Red 87 ligand solution. Analysis of the spectral data, presented in Fig. 4, revealed a distinct optimum for the analytical signal intensity. Maximum response was consistently achieved when employing volumes of the 0.03% (w/v) Acid Red 87 solution within the range of 0.2 to 2.2 mL. Consequently, a volume of 1.2 mL was selected for subsequent procedures as it represented the midpoint of this optimal range and ensured maximal reaction efficiency.

Deviations from this volume range detrimentally impacted the signal. Insufficient ligand volumes (below 1.0 mL) provided an inadequate concentration of Acid Red 87 to fully complex the available analyte, resulting in a diminished analytical response. Conversely, the data indicated that exceeding the upper limit of the optimal range (1.4 mL) also led to signal attenuation. This reduction at higher ligand concentrations is attributed to concentration-dependent self-aggregation of the Acid Red 87 molecules, a phenomenon visualized in Fig. 4. Such aggregation promotes non-radiative decay pathways, effectively quenching the ligand’s fluorescence reactivity and diminishing the observed signal for the ion-associate complex.

### Dispersing liquid impact

The influence of diluting solvent on the fluorescence quenching efficiency was systematically evaluated, as presented in Fig. 4. Several solvents were assessed, including acetone, distilled water, dimethylformamide (DMF), methanol, DMSO, ethanol, acetonitrile, and dioxane. Among these, distilled water (DW) yielded the most significant enhancement in the quenching response. This solvent also presents a distinct advantage from an environmental perspective, being the most sustainable option evaluated.

The diminished quenching efficiency observed with organic solvents is likely attributable to their detrimental effects on the electronic structure or stability of the reacting species. These solvents may interfere with the optimal molecular conformation necessary for complexation or perturb the electron distribution within the Acid Red 87 ligand, thereby hindering efficient ion-pair formation with PPX. Such interference can compromise the stability and yield of the PPX- Acid Red 87 complex. Consequently, distilled water was identified as the optimal solvent for preparing reagent solutions in subsequent analytical procedures.

### Validation of the system

The evaluation of factors like linearity, precision, sensitivity thresholds, accuracy, selectivity, and adaptability of the probe’s characteristics was carried out according to the standards established by the ICH^53^.

### The linearity and range

A standardized graph was constructed by graphing PPX concentrations against the mean RFI value, obtained from the system’s reaction to different PPX doses (in triplicate). The regression equation and associated statistical data were calculated utilizing the least-squares regression technique. The method demonstrated analytical linearity from 50 to 1400 ng/mL. A sensitivity analysis of the system was performed according to the LOQ and LOD formulas specified in the ICH recommendations. The detection limits were established at 3.3X/Y, and the quantification limits at 10X/Y, with ‘Y’ denoting the calibration slope and ‘X’ indicating the standard deviation of the intercept. The findings indicated that the Limit of Detection (LOD) and Limit of Quantification (LOQ) should be set at 15.2 and 46 ng/mL, respectively. All statistical factors employed in the investigating methodology for PPX are delineated in Table 1.

**Table 1:** Statistical data provided to substantiate the development of the syst.

Parameters	Values
Linear range (ng/mL)	50–1400
Slope	1.49
Intercept	388.23
SD of intercept (S_a_)	6.9
Correlation coefficient (r)	0.9994
Determination coefficients (r^2^)	0.999
Number of determinations	6
Limit of quantitation (ng/mL)	46
Limit of detection (ng/mL)	15.2

### Accuracy and precision

The provided approach was implemented across various concentrations of PPX, all falling within the calibration span. Three samples were analyzed for each PPX concentration to ensure robustness. The accuracy was confirmed through the percentage recovery estimates and standard deviation (SD). Results depicted in Table 2 revealed a significant alignment between predicted and actual values. Furthermore, three replicates were scrutinized at differing PPX concentrations to assess the precision of the strategy. Table 3 illustrates that the method exhibited high levels of precision at both examined levels, as evidenced by the low values of RSD, underscoring the reliability of the proposed methodology.

**Table 2:** The developed system’s accuracy at four PPX concentrations.

Conc. Level (ng/mL)	Recovery^*^± SD	% Error
200	101.27 ± 1.68	1.27
500	100.24 ± 0.67	0.24
800	99.25 ± 1.43	0.75
1100	98.07 ± 1.43	1.93

*Mean of three PPX determinations and SD is the standard deviation.

**Table 3:** Precision of the created methodology at two levels.

Conc. Level (ng/mL)	Intraday precision		Interday precision	
	Recovery^*^± SD	RSD	Recovery^*^± SD	RSD
150	98.93 ± 1.69	1.71	98.63 ± 1.86	1.89
600	100.79 ± 1.13	1.12	101.50 ± 0.55	0.54
1000	101.91 ± 1.55	1.52	100.07 ± 1.13	1.12

*Mean of three PPX determinations and RSD is the relative standard deviation.

### Robustness

Deliberate modifications were introduced to the experimental parameters, involving alteration to pH and the quantities of buffer, and Acid Red 87 for the investigation. The outcomes of these adjustments were analyzed, and the percentage recoveries along with standard deviations were computed, as detailed in Table 4. The minimal relative standard deviation values indicated that the method’s performance remained relatively stable, underscoring its consistent and acceptable robustness.

**Table 4:** Evaluating the robustness of the existing PPX assay technology.

Parameter	Value	% Recovery* ± SD
pH	3.7	99.14 ± 1.46
	3.9	100.01 ± 1.80
Buffer volume (mL)	1.2	98.86 ± 1.28
	1.4	99.14 ± 1.11
Dye volume (mL)	1.2	101.99 ± 1.17
	1.3	99.53 ± 1.88

*Mean of three PPX replicate measurements, SD = Standard deviation.

### Application

#### Tablet dosage form and content uniformity

Ramixole^®^ 1 mg tablets underwent a comprehensive analysis according to the specified approach’s procedure. The mean %recovery values were determined to be **98.92 ± 1.64**. To validate the results, a statistical comparison was conducted against the data obtained through the established fluorimetric method^15^, utilizing Student’s F- and t-tests. Interestingly, the calculated values of these tests were found to be lower than the tabulated ones, indicating a lack of significant variance in accuracy and precision between the provided and previously reported approaches, as illustrated in Table 5. When the content of the medication in the tablets falls below 25 mg or constitutes less than 25% of the tablet’s composition, it is advisable to assess the uniformity of the medication within the tablets. However, the procedure of inspecting each tablet’s content for consistency demands a considerable amount of effort and time. Therefore, the provided method presents notable advantages by being straightforward and expeditious, eliminating the need for time-consuming extraction or heating to instantly produce the complex. These benefits facilitate the direct evaluation of Ramixole 1 mg tablets. The data outlined in Table 6 illustrates that the homogeneity of Ramixole 1 mg tablets is affirmed, given that the acceptance value is below the maximum allowable threshold.

**Table 5:** Dosage form analysis of PPX and comparison with the reported method^15^.

Dosage form	Current method	Reported method		
	Recovery^a^ ± SD	Recovery^a^ ± SD	t- test value^b^	F-value^b^
Ramixole 1 mg tablets	98.92 ± 1.64	99.65 ± 1.93	0.64	1.39

^a^The value is the average of five determinations for both the proposed and reported methods.^b^ Tabulated values at 95% confidence limit are t = 2.306, F = 6.338.

**Table 6:** Application of the proposed methods for the content uniformity testing of PPX in Ramixole 1 mg tablets.

Tablet number	%Recovery
1	98.19
2	100.18
3	97.74
4	96.90
5	102.22
6	96.01
7	98.81
8	100.12
9	98.78
10	96.60
Mean ($\:\overline{X}$) ± S.D	98.55 ± 1.90
Acceptance value (AV)	4.56
Max. allowed AV (L1)	15

### Method greenness and whiteness

Green chemistry principles are increasingly vital in analytical chemistry. Defined by the Environmental Protection Agency (EPA) as the design of chemical products and processes that minimize hazards to human health and the environment, thereby preventing pollution, green chemistry guides the development of safer methodologies. A key strategy involves exploring non-toxic, renewable reaction media like water, ionic liquids, or supercritical fluids as alternatives to harmful solvents. This commitment underpins the development of the present green, environmentally safe, and rapid instrumental method for PPX determination^51^.

**Fig. 5:**
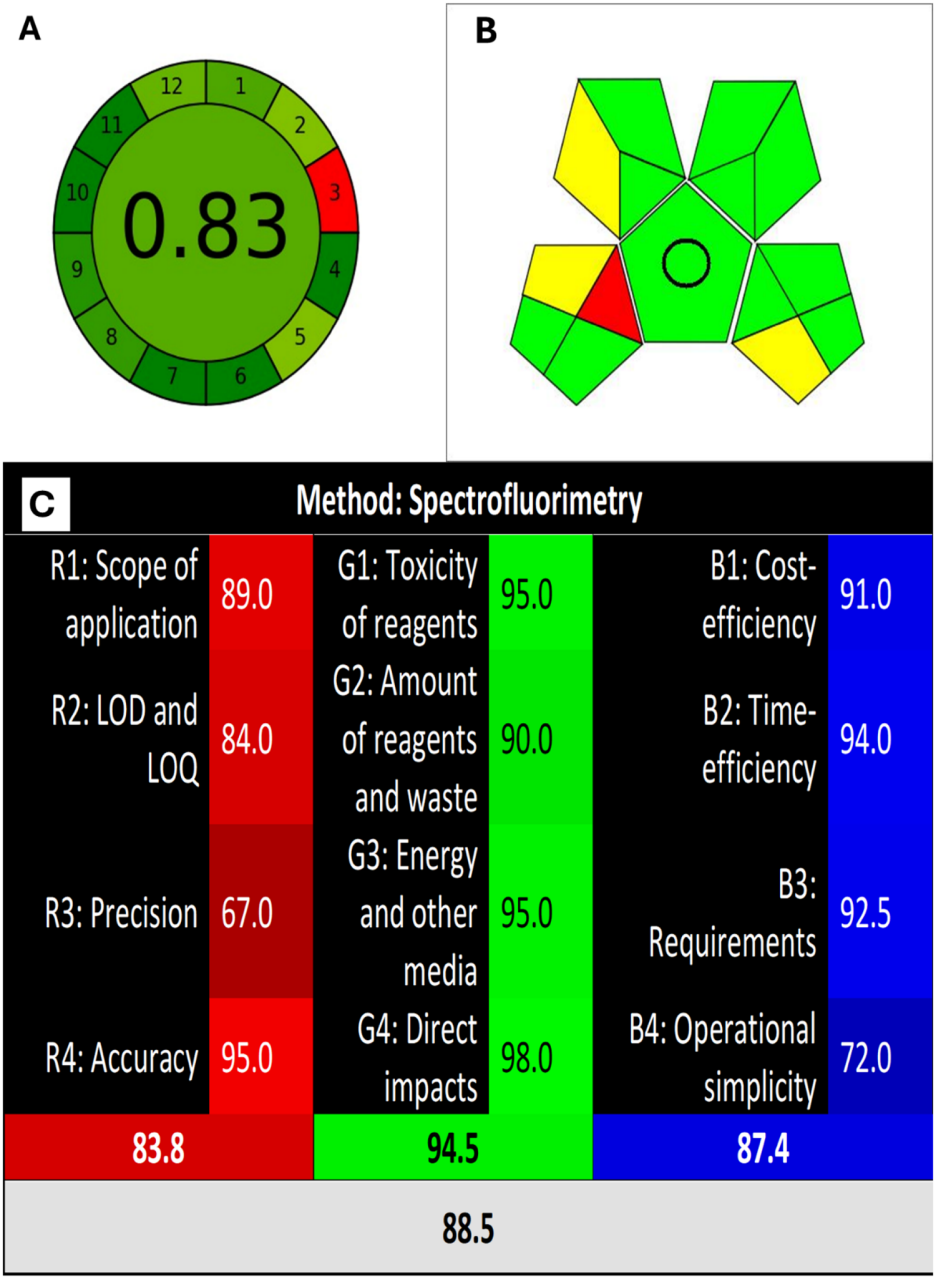
Representation of the method greenness using Agree (**A**) and GAPI tool (**B**) and RGB 12 (**C**).

The growing need to mitigate the environmental impact of analytical processes has spurred the development of specialized assessment tools. Conventional techniques, such as HPLC, are significant contributors to pollution, generating substantial organic waste (e.g., ~ 0.5 L per system daily). Consequently, evaluating a method’s greenness is now essential. Tools like AGREE provide a visual clock-shaped pictogram divided into 12 segments, each representing a Green Analytical Chemistry (GAC) principle, with a central numerical score (closer to 1 indicating lower ecological impact). As Fig. 5A illustrates, the proposed method achieves a highly favorable AGREE score. Similarly, the GAPI tool employs a five-stage pentagram system where color progression from green to yellow to red signifies increasing environmental impact across analytical steps. The proposed method demonstrates exceptional greenness for pharmaceutical analysis, primarily due to its complete avoidance of organic solvents. Further contributing to its environmentally friendly profile are the use of low-energy spectrofluorometric instrumentation, high sample throughput, and straightforward sample preparation that eliminates derivatizing agents, as detailed in Fig. 5B^54^.

Nowak and colleagues (2021) developed the innovative RGB12 algorithm as a comprehensive green assessment tool for analytical methods. This approach uniquely evaluates procedures across four distinct dimensions, visually represented by color-coded segments in its output. The red segment focuses on method validity, rigorously assessing performance characteristics including accuracy, precision, limits of detection (LOD) and quantification (LOQ), and overall analytical applicability. Environmental sustainability is gauged within the green segment, explicitly measuring adherence to the 12 core principles of Green Analytical Chemistry (GAC). The blue segment evaluates analytical productivity, considering metrics such as cost-effectiveness, practical implementation requirements, time efficiency, and operational simplicity. Finally, the white segment quantifies the holistic sustainability of the method, determined by its alignment with the 12 principles of White Analytical Chemistry (WAC), which emphasize the procedure’s overall balance and fitness for its intended analytical purpose. The RGB12 tool delivers a user-friendly graphical assessment through a freely accessible Excel worksheet that generates the results. As depicted in Fig. 5C, applying the RGB12 algorithm to the proposed method demonstrates its robust sustainability profile across all four evaluative dimensions^55–59^.

## Conclusion

In summary, this research successfully establishes and validates a novel, streamlined spectrofluorimetric assay for quantifying pramipexole (PPX) in pharmaceutical formulations. The method capitalizes on the rapid formation of a 1:1 ion-pair complex between protonated PPX and the Acid Red 87 anion under optimized acidic conditions (pH 3.8), resulting in a measurable quenching of the dye’s inherent fluorescence. This one-pot approach demonstrates significant analytical advantages, including exceptional simplicity by eliminating extraction or derivatization steps, cost-effectiveness through the use of readily available reagents and instrumentation, and rapid execution due to instantaneous complex formation. Rigorous validation according to ICH guidelines confirmed the method’s linearity (50–1400 ng/mL), sensitivity (LOD: 15.2 ng/mL, LOQ: 46 ng/mL), accuracy, precision, and robustness. Its successful application to the determination of PPX in Ramixole^®^ 1 mg tablets, yielding high recovery (98.92 ± 1.64%), and the efficient assessment of tablet content uniformity underscore its practical utility for routine quality control. Critically, the exclusive use of distilled water as the solvent aligns with green chemistry principles, further enhancing the method’s appeal as an environmentally benign alternative to existing chromatographic and spectrofluorimetric techniques requiring hazardous solvents or complex procedures. This Acid Red 87-based quenching strategy thus presents a reliable, efficient, and sustainable tool for PPX analysis.

### Data Availability

The datasets used and/or analyzed during the current study are available from the corresponding author upon reasonable request.

## Data Availability

The datasets used and/or analyzed during the current study are available from the corresponding author upon reasonable request.
